# TRAIT2D: a Software for Quantitative Analysis of Single Particle Diffusion Data

**DOI:** 10.12688/f1000research.54788.2

**Published:** 2022-01-31

**Authors:** Francesco Reina, John M.A. Wigg, Mariia Dmitrieva, Bela Vogler, Joël Lefebvre, Jens Rittscher, Christian Eggeling

**Affiliations:** 1 Leibniz-Institut für Photonische Technologien e.V, Jena, Germany; 2 Institute of Applied Optics and Biophysics, Friedrich-Schiller-Universität, Jena, Germany; 3 Department of Engineering Science, University of Oxford, Oxford, UK; 4 Département d'informatique, University of Quebec at Montreal, Montreal, Canada; 5 MRC Human Immunology Unit, Weatherall Institute of Molecular Medicine, University of Oxford, Oxford, UK

**Keywords:** Single Particle Tracking, Diffusion, Data analysis, Python, Graphical User Interface, Simulation, Microscopy

## Abstract

Single particle tracking (SPT) is one of the most widely used tools in optical microscopy to evaluate particle mobility in a variety of situations, including cellular and model membrane dynamics. Recent technological developments, such as Interferometric Scattering microscopy, have allowed recording of long, uninterrupted single particle trajectories at kilohertz framerates. The resulting data, where particles are continuously detected and do not displace much between observations, thereby do not require complex linking algorithms. Moreover, while these measurements offer more details into the short-term diffusion behaviour of the tracked particles, they are also subject to the influence of localisation uncertainties, which are often underestimated by conventional analysis pipelines. we thus developed a Python library, under the name of TRAIT2D (Tracking Analysis Toolbox – 2D version), in order to track particle diffusion at high sampling rates, and analyse the resulting trajectories with an innovative approach. The data analysis pipeline introduced is more localisation-uncertainty aware, and also selects the most appropriate diffusion model for the data provided on a statistical basis. A trajectory simulation platform also allows the user to handily generate trajectories and even synthetic time-lapses to test alternative tracking algorithms and data analysis approaches. A high degree of customisation for the analysis pipeline, for example with the introduction of different diffusion modes, is possible from the source code. Finally, the presence of graphical user interfaces lowers the access barrier for users with little to no programming experience.

## Introduction

Single particle tracking (SPT) is one of the most direct and employed methods to quantify particle dynamics in a sample using optical microscopy. As the name suggests, this approach relies on the identification and tracking of single particles in a sample, followed by the analysis of the detected trajectories. The analysis of particle trajectories is usually carried out using the description given by Einstein and Smoluchowski of Brownian motion ^1, 2^ to derive the diffusion coefficient of the objects. Given its nature as a data analysis method rather than a technique onto itself, SPT is indifferent to how the particle is detected, as long as the signal-to-noise levels are sufficient to identify it against the background. As a consequence of this, it is almost always necessary to label the sample with adequate target specificity, and it is important that the labelling should be sparse enough to enable single molecule level of detail, particularly when employing conventional, diffraction-limited microscopy. ^3^^,^^4^

Among the earliest example of SPT experiments, we can mention the testing of Einstein’s theory of Brownian motion, ^5^ during which tracking was executed by purely analog means, *i.e.* using pen and paper. The introduction of electronic digital camera detection and innovative microscopy systems have made it possible to extend the range of applications of SPT, which today reaches framerates up to 50kHz. ^6^ More importantly, the use of large scattering tags is nowadays not necessary anymore to reach nanometer levels of localisation precision. ^7, 8^ The rise of such SPT-capable techniques, characterized by fast sampling and high data throughput, thus generates the need to adapt previously developed tracking and analysis pipelines.

Conventionally, trajectory analysis in diffusive systems is performed by monitoring the Mean Squared Displacement of the particle against the time increments, and analyzing them through the Brownian motion ^1^ and similar models of diffusion. ^9, 10^ While this kind of pipeline has the advantage of being straightforward and easy to implement, it is not immune from potential pitfalls. As we elaborate further in the Methods section, the localisation error is a critical factor that is often overlooked. ^11^ This aspect has become especially critical in recent times, given the rise in sampling rates accessible with modern hardware. Motion blurring, which is inherent in SPT given the movement of the target particles, is also often neglected as a potential source of measurement error, ^12^ which can also lead to disruptive effects in the estimation precision of relevant physical parameters. Overlooking these sources of error leads to overestimation of the detected diffusion coefficients, ^13^ or to erroneously detected subdiffusion. ^14^ These spurious deviation arise particularly at short time ranges, given that the particle displacements at these time frames is comparable to the localisation noise, and is thus particularly dangerous since many analysis pipelines often rely on the very first data points to extract relevant diffusion parameters. ^15^ In addition to this, another factor that affects the correct estimation of physical quantities is the model fitting methodology employed and the number of Mean Squared Displacement points to employ in the data analysis pipeline, which require careful consideration on an ad-hoc basis for each dataset. ^16^^–^^18^

Numerous toolboxes and algorithms have been produced to fulfill the need to identify and track single particles, ^19– 21^ and analyse their motion employing conventional mean squared displacement analysis, ^22^machine and deep learning ^23– 25^ and other methods. ^26, 27^ Oftentimes the source code is shared by the authors and is readily available, and while in some cases they are not platform-agnostic and require licensed software to be executed, on the other hand, e.g., web-based tools and open source alternatives have also been developed to widen the access possibility to such tools. On the other hand, tools available on open-source platforms, such as FIJI ^28^ or ICY, ^29^ are often more focused on the particle tracking, rather than the data analysis itself.

The TRAIT2D (Tracking Analysis Toolkit - 2D version), hereby presented, aims to supply the scientific community with an accessible, open-source, and platform-agnostic tool for particle tracking, simulation and analysis in two dimensions. In TRAIT2D, intuitive graphical user interfaces facilitate users without coding background to promptly access the tools. The tracking tool provided is the most specialised part of the package, as it was developed for particle tracking techniques with a high sampling rate, capable of acquiring long, uninterrupted trajectories, such as Interferometric Scattering (iSCAT). ^30^ Thereby, the trajectory linking is based on the algorithm that favour strong spatial and temporal connections between consecutive frames. The choice of particle localization algorithm, based on the radial simmetry approach, ^31^ is linked with the fact that the data which guided the development of the tool features symmetrical, round particles diffusing on a plane.The analysis and simulation tools, on the other hand, have a wider scope of applications. Once the particle trajectories are obtained, either via the provided tracking tool, or imported from other sources, two avenues are available to perform single particle trajectory analysis. The first and more widely adopted, is through the Mean Squared Displacement, which many users will find more familiar and is still conventionally employed. The second avenue, which we named Apparent Diffusion Coefficient analysis, provides more insights into the localisation error and a more intuitive way of monitoring the diffusion behaviour at different time scales. A statistics-driven assists the user in finding the most appropriate diffusion model to describe the particle trajectories. The source code allows more advanced users to customize data analysis, allowing, for example, the introduction of user-defined diffusion models that integrate seamlessly with the proposed analysis pipeline. The track simulator allows the user to generate particle trajectories on a homogeneous plane, or on a surface divided in compartments where the particle can be transiently confined, with a high degree of customisation. We also added the possibility of generating movies from the simulated trajectories with variable levels of contrast, and a user-specified Point Spread Function. This last module can serve as a validation tool of further particle tracking and analysis pipelines.

## Methods

### Particle tracking

The particle tracking is developed to support a quantitative analysis module which requires extracted trajectories. We introduce a tool which allows users to adjust tracking parameters, preview results and save final trajectories.

The pre-processing of the image sequence is optional and can be applied prior to the tracking algorithm. The tracking is implemented as a two-step process. Firstly, the particles are detected using spot enhancing filter (SEF) ^32^ and sub-pixel localisation is estimated by the radial symmetry centre approach. ^31^ Secondly, the detected particles are linked based on their spatial and temporal locations.

The current tool was developed for the iSCAT imaging technique and the pre-processing step is built in accordance to the technique requirement. To distinguish the molecules of interest from the background, the image sequence is divided by a flat field. The flat field represents an undesired static background scattering, and it is calculated by the pixel-wise temporal median filter over a set of frames (1000 frames in the current version). The background is calculated as a temporal average of the entire image sequence and subtracted from each frame. At last, the movie is normalized and brightness adjustment is applied to the image sequence. ^33^ Although the pre-processing step is developed to subtract a temporal median filter, as it is conventionally done in iSCAT microscopy, it can also be employed for other techniques as a background subtraction tool.

The particle detection exploits a spot enhancing filter (SEF) to enhance the particles and reduce correlated noise in the image. The SEF can be described by the convolution of the original image with a Laplacian-of-Gaussian (LoG) kernel followed by global thresholding to extract the spots.

f(x,y,σ)=LoG(x,y,σ)*g(x,y), (1)

where *σ* is a standard deviation of the LoG. The LoG is a second-order derivative filter, and is commonly used as a base for spot detection. ^34^ The solution can be adjusted for a different scale by altering *σ*. The threshold is defined by the average intensity *μ*_intens_ of the image and its standard deviation *σ*_intens_, weighted by the constant *c*:

$$T=\mu_{\text{intens}}+\sigma_{\text{intens}}$$ (2)

The constant *c* is related to the spot intensity, and together with *LoG* parameter *σ* can be adjusted by the user. The local maximum of the image identifies a set of the detected particles in each frame. Further refinement of the particle coordinates (sub-pixel localisation) is implemented with the radial symmetry centre approach. ^31^ It provides a faster execution time due to its non-iterative nature, while achieving high accuracy. The size of the region of interest for the sub-pixel localisation and the limitation for the detected peak size can be set manually. The radial symmetry centre approach is limited to a symmetrical round particle, and therefore, for a case of different particle shape, the sub-pixel localisation function has to be replaced with an alternative solution, for example. ^35– 37^ Furthermore, the user should note that this algorithm only provides the centroid positions of the detected particles, and does not feature a precise determination of the standard deviation of the fitted 2D gaussian function, or other parameters such as integrated intensity.

The iSCAT imaging technique provides high temporal resolution. The small displacement of the particle between neighbouring frames allows reducing complexity of the linking algorithm and increase the computational speed. The linking approach focuses on strong spatial connection between frames. Hungarian combinatorial optimisation algorithm ^38^ is employed for the task of data association. The tolerance of the algorithm towards the spatial and temporal distance can be set by the parameters in the graphical-user interface. The assembled tracks can contain gaps in temporal domain due to the failed detection in one or few frames. These gaps are filled taking into account detections in the neighbouring frames and sub-pixel localisation. At the final stage, all the trajectories are filtered based on their length.

### Mean squared displacement analysis

In this and the following section, we will briefly discuss the analysis methods implemented in the TRAIT2D software package. The first method, which we refer to in the code as “MSD analysis”, consists of the approach to determine the physical parameters of diffusion dynamics based on the behaviour of the mean squared displacements against the corresponding time interval.

In the case of MSD analysis, the MSD is initially calculated according to the definition:

$$MSD(t_{n})=\frac{1}{N-n-1}|\underline{r}(t_{i+n})-\underline{r}(t_{n}){|}^{2}$$ (3)

whereby *t _n_* = *nt*_0_ indicates the n-th time point from the initial time *t*_0_, and $\underline{r}\left(t_{n}\right)$ indicates the two-dimensional position vector at time *t _n_.* Notice that this notation, as well as the entire analysis pipeline hereby implemented, supposes that the time interval between localisation is constant. According to Einstein’s theory of Brownian Motion generalized for higher dimensionality, ^1, 4^ this quantity is linked to the diffusion coefficient, *D*, and time by the formula:

$$MSD(t_{n})=2dDt_{n}$$ (4)

in which *n* indicates the dimensionality of the system. In the rest of the section, we will assume *d* = 2 to simplify the formalism. In order to account for experimental finite localisation precision, it is necessary to introduce an additive term to 4 ^39^:

$$MSD(t_{n})=4D(t_{n})t_{n}+2\delta_{x^{2}+y^{2}}^{2}.$$ (5)

The above formulation assumes that the localisation error in the *x* and *y* directions is identical. This formula has been generalized to include the possibility of anomalous subdiffusion:

$$MSD(t_{n})=4D(t_{n})t_{n}^{\alpha }+2\delta_{x^{2}+y^{2}}^{2}.$$ (6)

While the user can, of course, fix the localisation error value according to their chosen metric, it is also possible to leave it as a floating parameter. This is theoretically preferable from the point of view that the detection of moving particles is by necessity less precise than that of immobile particles, which are often used as a reference to estimate the localisation precision of a microscopy system. The effect of motion blurring, to which this loss in precision is ascribable, is represented by a negative term by the form ^11^:

$$\delta_{Blurring}=-8DRt_{lag}$$ (7)

where *R* is a term which depends on the illumination mode of the chosen microscopy technique, and obeys 0 ≤ *R* ≤ 1/4. It is especially interesting to notice how this term is dependent not on the time variable, but rather to the time resolution of the measurement. As a consequence of this, non-blur-corrected MSD data may suffer from considerable distortion at short time intervals, which coincidentally are the ones that are considered most important in determining the kind of motion the particle undergoes. ^12, 40^ When this term is added to Eq. 5 and Eq. 6, it gives rise to the formulas:

$$MSD(t_{n})=4D(t_{n})t_{n}+2\delta_{x^{2}+y^{2}}^{2}-8DRt_{0}$$ (8)

and

$$MSD(t_{n})=4D(t_{n})t_{n}^{\alpha }+2\delta_{x^{2}+y^{2}}^{2}-8DRt_{0}$$ (9)

which are ultimately used to perform curve fitting and extract the relevant parameters. In the case of free diffusion ( 5 and 8), we once again remark the necessity to consider an adequate range of data over which to set up the model fitting routine, as highlighted in the ntroduction. The same observation, for the same model, is also valid if the user elects to use the following Apparent Diffusion Coefficient analysis.

### Apparent diffusion coefficient analysis

The apparent diffusion coefficient (ADC) analysis differs from the MSD analysis mainly for the central role given to the diffusion coefficient compared to mean squared displacement. We define the ADC as:

$$D_{app}(t_{n})=\frac{MSD(t_{n})}{4t_{n}\left(1-\frac{2R}{n}\right)}$$ (10)

thereby enabling us to switch to a diffusion coefficient-centric representation. The nomenclature ”apparent” is adopted to stress how this quantity is still affected by the localisation error and not the absolute value of the diffusion coefficient. Dividing both terms of Eq. 8 by $4t_{n}\left(1-\frac{2R}{n}\right)$ , and with some elementary algebra, it is possible to derive:

$$D_{app}(t_{n})=D(t_{n})+\frac{2\delta_{x^{2}+y^{2}}^{2}}{4t_{n}\left(1-\frac{2R}{n}\right)}.$$ (11)

By substituting in place of *D*( *t _n_*) the appropriate expression for the diffusion coefficient for a specific model, it is therefore possible to obtain a complete model to fit to the apparent diffusion coefficient data, obtained according to Eq. 10. In principle, it is possible to leave the localisation error $\delta_{x^{2}+y^{2}}^{2}$ as a free-floating parameter in the fitting operation, to better estimate the localisation error for moving particles.

The software package hereby presented comes with three built-in diffusion models, ^12, 41, 42^ corresponding to free (Brownian) diffusion

$$D(t_{n})=D_{M},$$ (12)

confined diffusion

$$D(t_{n})=D_{\mu }\left(\frac{\tau }{t_{n}}\right)\left(1-e^{-\frac{\tau }{t_{n}}}\right),$$ (13)

and compartmentalized diffusion, which is the combination of the above two models

$$D(t_{n})=D_{M}+D_{\mu }\left(\frac{\tau }{t_{n}}\right)\left(1-e^{-\frac{\tau }{t_{n}}}\right).$$ (14)

The nomenclature for these models follows the convention that *D _M_* is a constant diffusion coefficient, corresponding to theoretical Brownian diffusion, and that *τ* is the characteristic residence time in the confinement zones described by the confined diffusion model. *D _μ_* is the microscopic diffusion coefficient observed in the aforementioned confinement zones, which can also be expressed as

$$D_{\mu }=\frac{L^{2}}{12\tau }$$ (15)

where *L* is the average confinement zone size. ^10^ It is apparent that while this data analysis pipeline appears more complex, it however readily provides an efficient way to directly extract relevant physical parameters from the particle trajectories. We point out that, although the code we hereby describe comes with the aforementioned built-in models, chosen according to the authors specific research interests, a documented and simple procedure is in place to define a new model, according to the end user-specific wishes. The only restriction is that the custom-defined models can only be used when the analysis module is imported in a Python script. However, the analysis pipeline will integrate this addition seamlessly alongside the other models, or run exclusively on the user-defined models.

The selection of the best model to describe any given track is done by statistical means. Each of the models is fitted to the *D _app_* data, and the Bayesian information criterion (BIC) for each model is calculated according to the formula ^43^:

BIC=k ln(n)+n ln(RSS/n) (16)

in which *n* is the number of data points the model is fitted to, the RSS is the residual sum of squares, and *k* is the number of degrees of freedom, i.e. the free parameters, of the fit. The most adequate model to describe the diffusion motion is then the one with the lowest value of BIC. Since the BIC is not a goodness-of-fit metric, the analysis function will also perform a one-sided Kolmogorov–Smirnov test, comparing the original ADC data against the results of the fit.

### Simulation tools

In order to validate the analysis methods described above, we have developed a simple simulation framework. Our tool can create synthetic particle tracks using various diffusion models. Furthermore, the tracks can be converted into synthetic movies to test the particle detection method.

### Track simulation

The free (Brownian) diffusion was simulated by drawing a random walk on a two-dimensional virtual square space of side *L*, entered by the user. The particle is initialized in the centre of the square. The user also has the ability to select the value for the diffusion coefficient *D* and the time step *dt.* The position of the particle is updated at each successive time step in the *x* and *y* direction separately:

$$\begin{array}{ll} x(t+dt) & =x(t)+\sqrt{\left(2*D*dt\right)}*(\text{rand})\\ y(t+dt) & =y(t)+\sqrt{\left(2*D*dt\right)}*(\text{rand}) \end{array}$$

This set of equations does not strictly respect the condition that ( *x*( *t* + *dt*)− *x*( *t*)) ^2^ + ( *y*( *t* + *dt*)− *y*( *t*)) ^2^ = 4 * *D* * *dt*, since doing so would not account for the variability that happens in real simulations. Instead, the theoretical displacement $\sqrt{D*dt}$ is multiplied by a random number (“rand“) extracted from a normal distribution of mean *μ* = 0 and *σ* = 1.

The hop diffusion motion was simulated according to the code used for, ^44^ kindly provided by Dr. J. Keller–Findeisen as a Matlab script, and transcribed into Python by the authors. First of all, a virtual surface of arbitrary dimension is split into an arbitrary number of compartments as a Voronoi diagram with random seed. To each of the compartments, a unique identifier is assigned. A particle is then generated in the centre of the plane, and its motion is simulated in the same way as the Brownian diffusion case. However, an additional condition is in place to simulate the hopping motion. A probability is assigned to the particle of jumping between one compartment and the other, called hopping probability ( *HP*). Every time that the randomly generated displacement would move the particle to a different compartment, a random number between 0 and 1 is extracted. Should this number be less than the value of *HP* initially fixed, the displacement calculation is repeated in order to keep the particle in the same original compartment.

Confined diffusion can easily be simulated by fixing *HP* = 0.

### Movie generation

We have created a simple movie simulator to obtain a synthetic movie from a list of detected or simulated tracks. This simulator is inspired by a similar noisy holographic image simulator. ^45^ Apart from a mandatory list of tracks to simulate, other, parameters required for the simulation are the spatial ( *r _xy_*) and temporal ( *r _t_*) resolutions, the contrast C = Ip − μbg between the simulated particle intensity Ip and the background signal level ( *μ _bg_*), and the Gaussian noise level ( $\sigma_{bg}^{2}$ ). Optional inputs are a point-spread function (PSF) stack, which can either be obtained experimentally or estimated with a model of the microscope, such as with DeconvolutionLab2. ^46^

The simulator is initialised using the given tracks. The number of time frames, the minimum, and the maximum spot positions in *X* and *Y* are extracted from all tracks. These are used to initialise the simulation grid. The ( *x _min_*, *y _min_*) and ( *x _max_*, *y _max_*) positions define the simulation grid width and height. If the grid needs to be square, the min and max between ( *x _min_*, *y _min_*) and ( *x _max_*, *y _max_*) are computed respectively to define the grid shape. The simulation grid is discretized using the spatial resolution *r _xy_.* For example, a track whose minimum position is (0,0), and maximum position is (10,10), and for a simulation resolution of *r _xy_* = 1, will have a shape of 11 × 11, where each grid position represents a pixel of size 1 × 1 μm ^2^. Furthermore, if the maximum time frame contained in a tract is 10, and if the temporal resolution is set to *r _t_* = 1, the simulated movie will be of shape (11 × 11 × 11), where the first two dimensions represent the spatial dimension, and the last is the temporal dimension.

Once the simulation grid is initialised, we first add a background signal $B(x,y,t)∼N(\mu_{bg},\sigma_{bg}^{2})$ everywhere. Each pixel background intensity follows a normal distribution of mean μbg and of variance $\sigma_{bg}^{2}$ , and each simulated background pixels are independent and identically distributed (i.i.d.) random variables. In other words, at this stage there is no spatial or temporal correlation between the pixels intensities. We are using the util.random_noise method from the scikit-image Python module. ^47^

Next, for each spot position in each track, we discretize the position in both the spatial $\left(x,y)\to (m_{x},m_{y}\right)$ and temporal dimensions $\left(t)\to (m_{t}\right)$ using

$$m_{i}=\left[\frac{x_{i}-x_{i,min}}{r_{i}}\right],$$ (17)

where *i* ∈{ *x,y,t*} represents the *x*, *y*, and *t* dimension of the spot positions respectively, *x _i_* is its position as recorded in the tract, *x _i,min_* is the minimum position within the tract, *r _i_* is the simulation resolution along the *i ^th^* axis, and [⋅] represents the rounding, operation to obtain discrete positions *m _i_* within the simulation grid coordinate framework. Each spot position is added iteratively to the simulated movie, and its intensity is given by the desired contrast *C* for this simulation. At this stage, the simulated movie can be described by the equation

$$M(x)=(1+SNR)\delta (x-m_{i})+B(x),$$ (18)

where *δ*( **x** − **m**_*i*_) is a n-dimensional Dirac delta function, x = ( *x*, *y*, *t*) is a (3,1) vector representing the spatiotemporal position within the simulation grid, **m** *_i_* = ( *m _x,i_*, *m _y,i_*, *m _t,i_*) is the discretized vector position within the simulation grid of the *i ^th^* spot, and *N* is the total number of spots among all tracks to simulate. If a PSF is given as input, it will be applied at this stage with a convolution operator. If a 3D PSF stack is given, the central slice of the stack (corresponding to the focus position) will be used instead of the whole 3D stack for the simulation. The PSF can either be an experimentally acquired stack or a simulated, for example, using the external DeconvolutionLab2 plugin for FIJI/ImageJ with the microscope’s configuration. ^46^ To consider the boundary effects, we use zero padding. Thus, the simulated volume is

$$M(x)=PSF(x,y)⊗\left[\left(1+SNR)\delta (x-m_{i})+B(x\right)\right],$$ (19)

where ⊗ is the convolution product. Finally, i.i.d. Poisson noise is simulated for each pixel separately, using the value of M(x) as the Poisson distribution parameter. The Poisson noise is simulated using the python module random_noise from scikit-image. Thus we obtain

$$M^{\prime }(x)∼P(k;\lambda (x)=M(x)),$$ (20)

where

$$P(k;\lambda )=\frac{\lambda e^{-\lambda }}{k!}$$ (21)

The simulated movie is exported as a tiff stack or an avi file. Figure 3 is a static example of a simulated movie frame using a single simulated track. The image on the left is a movie frame before the PSF convolution and the Poisson noise simulation, and the image on the right is the final simulated movie frame after these operations.

### Implementation

TRAIT2D is implemented entirely in Python using available packages.

The software components (simulation, tracking, analysis) have been split into separate modules that have graphical user interfaces (GUIs) available or, in the case of the simulation and analysis libraries, can be imported in Python scripts.

TRAIT2D uses an object-oriented approach for its workflows:

The simulation library uses Diffusion objects which hold the parameters and mathematical representation of a diffusion model. Specific models inherit from the base Diffusion class. Simulation results are stored in the simulator as a Python dictionary.

In the analysis library, tracks are represented as Track objects, which can be created from such a dictionary, a Pandas data frame or a CSV file. Multiple tracks can be pooled in a ListOfTracks object. Analysis is executed by calling the corresponding methods on these objects and results are stored in them. Central to the analysis library is, of course, the .msd_analysis() and .adc_analysis() methods of the Track and ListOfTracks classes. Through these methods, the user can fit a range of models (included with the library, or custom) to their data. The model fitting itself is ultimately performed using the .curve_fit() function of the scipy.optimize module, which uses a nonlinear least squares algorithm to fit the model function to the data. This function returns the optimal value of the parameters, and the relative covariance matrix. The square root of the diagonal elements of said matrix are thus taken as estimator for the fitting error of each parameter.

A unique property of the analysis library is the ability to define and add new diffusion models, through a “singleton” class called ModelDB. This functionality is only exposed when importing the analysis module from a Python script but not from the GUI.

### Operation

TRAIT2D requires at least a Python 3 (recommended ≥ 3.7) to run. The software also depends on additional packages: Graphical user interfaces are provided through PyQt (analysis) and Tkinter (simulation, tracking). Scipy, matplotlib and pandas are used for model fitting, plotting and loading data, respectively. These and other dependencies are automatically installed when installing from source using the supplied setup.py file or from the Python Package Index (PyPI).

## Use cases

This section outlines basic use cases for TRAIT2D. The complete documentation as well as a list of tutorials as available at https://eggeling-lab-microscope-software.github.io/TRAIT2D/.

### Workflow

**Figure 1.**
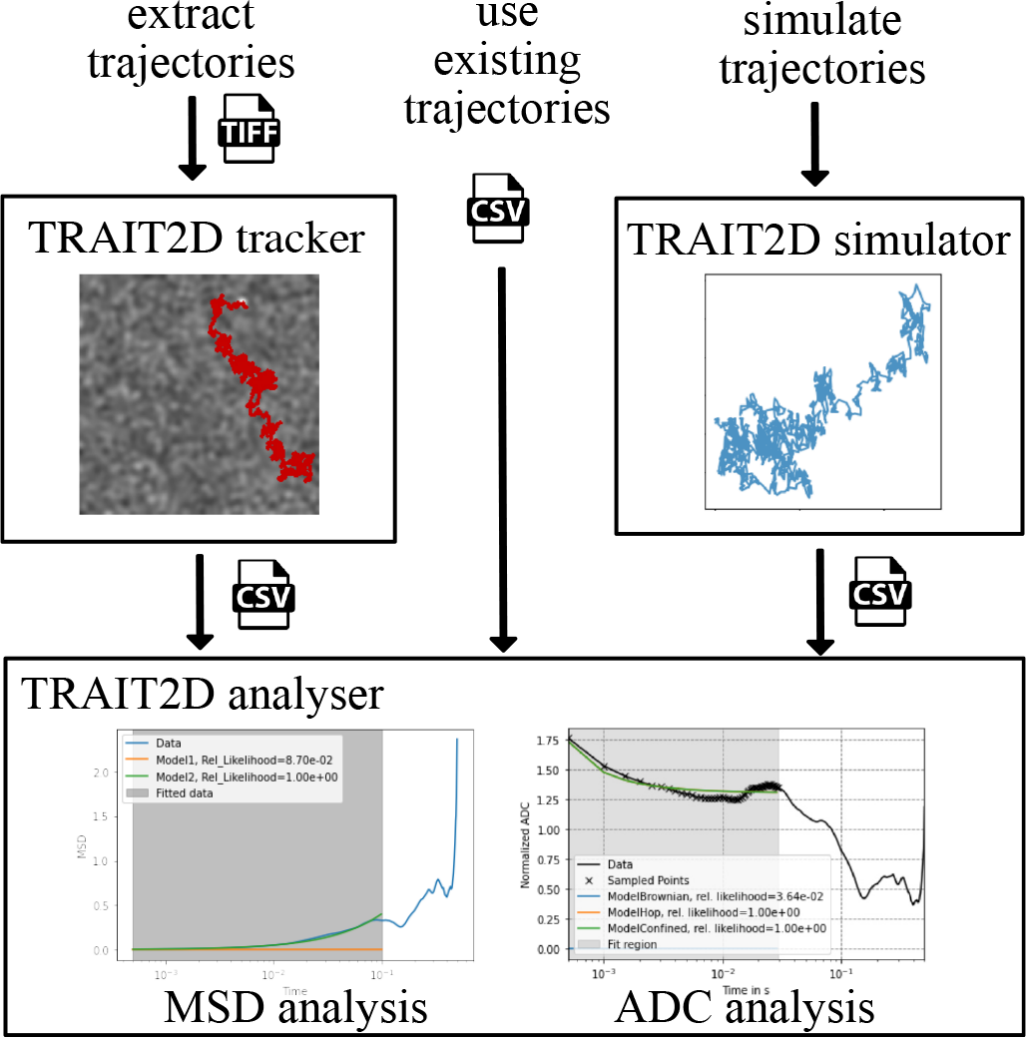
Schematic of the workflow of the TRAIT2D software package, highlighting the three possible paths to feed data to the analysis module: ( *left*) through the tracker, starting from a TIFF stack of the diffusing particles, *center* importing trajectories otherwise obtained as a CSV file, ( *right*) or by simulating particle trajectories with the simulator module, starting from user-defined parameters.

The central component of the TRAIT2D software package is the analysis module, through which it is possible to extract physical parameters from the particle trajectories. There are three possible avenues for the user to analyse their data ( Figure 1). By using the tracker module, the user can import their timelapses, as TIFF stacks, into the GUI and extract particle trajectories, which can then be exported as a formatted CSV file, ready to be imported in the analysis GUI. Alternatively, if the trajectories are already available to the user through a different particle tracking tool, they can be imported as well, on condition that their formatting is compliant with the format required by the analysis module. Finally, the user could also generate a control dataset through the simulator module, either through the GUI or as a script. The simulated trajectories will then be exported as a properly formatted CSV file, or a compliant data structure in the case of the user script. The Analysis GUI will perform the data analysis following either the MSD or the ADC pipelines (see Methods section), as indicated by the user. We stress that while the GUI exclusively uses the predefined set of models (see Eq. 12, 13 and 14) for the analysis, the module itself allows the addition of more diffusion models as well as batch processing when used in custom scripts, adding an ulterior degree of flexibility.

### Compatible file formats and data structures

The analysis library and GUI expects track data to be supplied in a specific format although column names and units are adjustable. At the time of the first release, the trajectories can only be imported from CSV files. A track file must include at least three columns, which contain the localisation time t as well as the coordinates x and y. The order in which these columns appear is not important. A unique numerical identifier id must be added as a fourth column in files which contain multiple tracks. This identifier will match each localisation with a specific track.

When working with the analysis library as an imported module inside a Python script, tracks can also be directly loaded from a dictionary or a Pandas data frame as long as they contain the keys described above.

### Installation

The package containing the full software can be downloaded directly from PyPI, using the command:



pip install trait2d


Alternatively, the source code is available at the Github repository:

https://github.com/Eggeling-Lab-Microscope-Software/TRAIT2D.

### Graphical user interfaces

Once installed, the different GUIs can be launched by entering trait2d_analysis_gui, trait2d_simulator_gui, or trait2d_tracker_gui in a Python-enabled environment.

### Importable modules

The analysis and simulation libraries can be used as modules in Python scripts by importing either trait2d.analysis or trait2d.simulators. This exposes additional features such as bulk analysis and adding custom diffusion models and allows the user to program pipelines for their specific use case. The tracker software is only available as a GUI. 22

### Tracking particles

**Figure 2.**
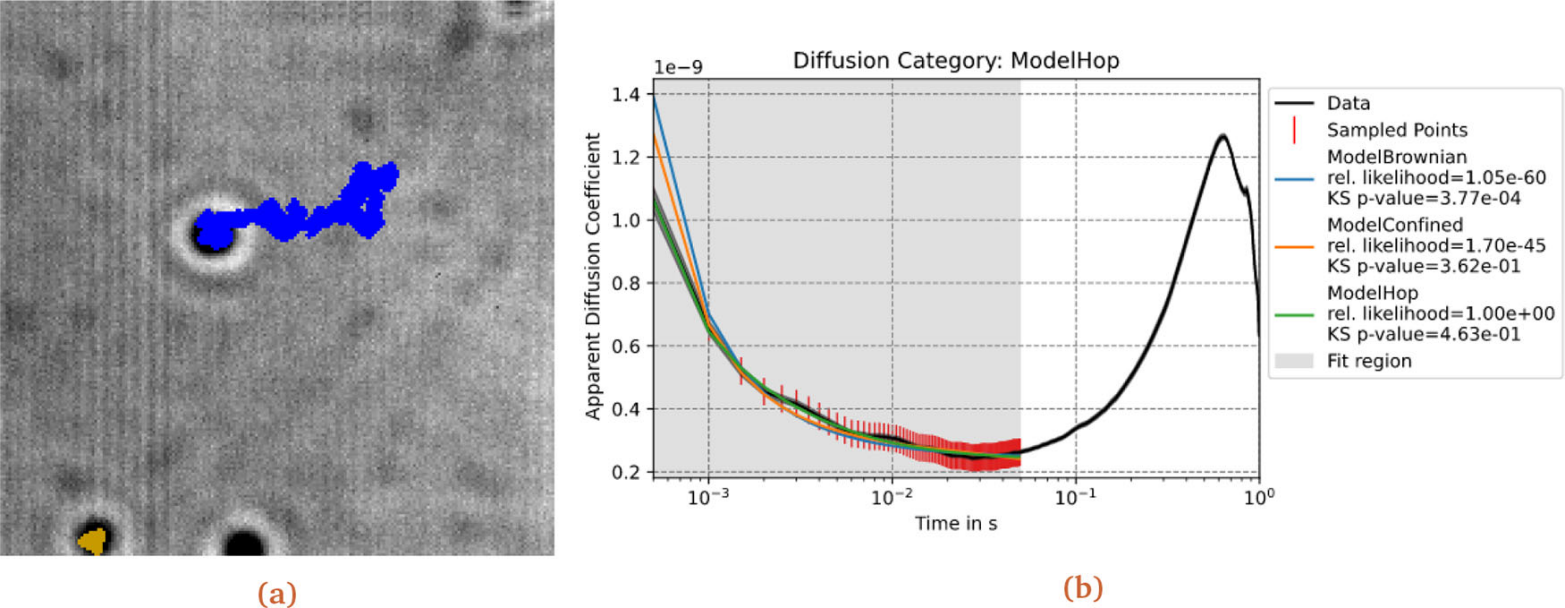
Processing a timelapse image. (a) Example of the output TIFF file, exported by the program after the tracking procedure has been completed. The tracker has found three tracks: one over the full movie length (blue) and a shorter track confined to the sport in the bottom-left corner (yellow). The latter is cut off due to the spot being close to the border. (b) Graphical result of the analysis, through the ADC pipeline, of the track in blue, using the three built-in models, with an opportune analysis interval, initial values and bounds for the parameters.

The particle tracker is available only as a GUI tool at the time of writing. It can be accessed by typing the command trait2d_tracker_gui in a terminal with a compatible Python installation. The application accepts 8 bit TIFF stacks as an input. It is possible to run a simple pre-processing step with background subtraction. The tracker GUI provides options to set parameters, and to preview particle detection results frame by frame. Once the parameters for detection and trajectory linking are deemed satisfactory by the user, the application will perform the trajectory linking over the image stack, and export another tiff stack where the detected trajectories are superimposed as a coloured track over the original data ( Figure 2). The user can thereafter choose to export the particle tracks as a csv file, with formatting compatible to the analysis tool.

### Analysing a track through the MSD pipeline

While the majority of the use cases presented here will be focusing on the more elaborate ADC analysis pipeline, a quick overview of the MSD analysis pipeline will be given in this section as well.

MSD analysis uses only the built-in linear (Eq. 8) and power (Eq. 9) models for fitting, and there is no option to add custom models.

Other features, such as bulk processing, however, are also available for the MSD pipeline and work similar to their ADC counterparts.

Below is an example illustrating the process of importing a single track from a file and running MSD analysis on it:



from trait2d.analysis import Track
single_track = Track.from_file("track1.csv")
single_track.msd_analysis(fit_max_time=0.5e-1)


Once again, we reiterate that the fit_max_time parameter, i.e. the time range over which the MSD analysis is performed, must be chosen with care to avoid misrepresenting the dynamics of the tracked particles, especially in the case of Brownian motion. ^16– 18^

### Analysing a track through the ADC pipeline

In order to be analysed, a track must be contained in a compatible file or data structure (see above). Data analysis can then be performed through either the analysis GUI, accessible by typing trait2d_analysis_gui in a terminal with a compatible installation of Python, or through a user script by importing the trait2d.analysis module. For the use of the analysis GUI, we would reference the documentation https://eggeling-lab-microscope-software.github.io/TRAIT2D/analysis_gui.html, where it is extensively described. The following code snippets exemplify how to load a single track from one of the example files provided with the code, and analyse it using a Python script.

First of all, the track data needs to be imported as a trait2d.analysis.Track object:


from trait2d.analysis import Track

single_track = Track.from_file("track1.csv")


In order to proceed with the analysis, the models need to be defined. The package comes with three default models (Eq. 12, 13 and 14), which can be handily registered in the trait2d.analysis.ModelDB object. All models added to this singleton object will then be used to analyse the track data. Lower and upper bounds, as well as initial values, can also be easily set on a per-model basis. The following code snippet shows, as an example, how to import the built-in brownian diffusion model (Eq. 12), and define the bounds and initial values necessary for the fitting operation:


import numpy as np
from trait2d.analysis.models import ModelBrownian
from trait2d.analysis import ModelDB
ModelDB().add_model(ModelBrownian)

ModelDB().get_model(ModelBrownian).initial = [0.0, 0.0]
ModelDB().get_model(ModelBrownian).upper = [np.inf, np.inf]
ModelDB().get_model(ModelBrownian).lower = [0.0, 0.0]


For the other models, the procedure is identical, however more or less parameters should be initialized, as required by their expression. Once the ModelDB has been populated with the desired diffusion models, the analysis is performed via the command: DM,

results = single_track.adc_analysis(fit_max_time=0.5e-1)


The analysis module saves the analysis results to the trait2d.analysis.Track object, so they can easily be accessed at a later point. More options concerning the whole analysis pipeline are also available in the documentation. Several examples are also provided as downloadable Jupyter Notebooks.

### Adding a custom model for ADC analysis

The analysis module allows to define custom diffusion models which can be used in the analysis pipeline, and integrate seamlessly into it. This is done by defining a class which inherits from trait2d.analysis.models.ModelBase. This functionality is only available when importing the module in a Python script and not from the GUI.



from trait2d.analysis.models import ModelBase
import numpy as np

class MyModelBrownian(ModelBase):
  lower = [0.0, 0.0]
  upper = [np.inf, np.inf]
  initial = [0.5e-12, 2.0e-9]

  def __call__(self, t, D, delta):
    return D+delta**2/(2*t*(1-2*self.R*self.dt/t))


The newly defined model can be added to trait2d.analysis.ModelDB, in addition to or instead of the built-in models and will be used in all subsequent analyses. This functionality is only available when importing the module in a Python script and not from the GUI.

### Bulk processing

When using the analysis module inside a Python script, bulk processing of multiple tracks is available. For this, the trait2d.analysis.ListOfTracks class is used. In the following example, a CSV file containing multiple tracks is loaded. In addition to MSD and ADC analysis, ListOfTracks also exposes useful methods such as ListOfTracks.plot_trajectories or ListOfTracks.adc_summary to quickly visualise all contents and analysis results of the contained tracks. Bulk processing is also available for MSD analysis *via* ListOfTracks.msd_analysis.



from trait2d.analysis import ListOfTracks
tracks = ListOfTracks.from_file("three_tracks.csv", unit_length='micrometres')

tracks.plot_trajectories()

from trait2d.analysis.models import ModelBrownian, ModelConfined, ModelHop
from trait2d.analysis import ModelDB

ModelDB().add_model(ModelBrownian)
ModelDB().add_model(ModelConfined)
ModelDB().add_model(ModelHop)

tracks.adc_analysis(fraction_fit_points = 0.15)
tracks.adc_summary(plot_dapp=True, plot_pie_chart=True)


### Simulating a track

Track simulation can be done either from the GUI, which can be accessed by typing trait2d_simulator_gui in a terminal with Python available, or by importing a simulator from trait2d.simulators.

The following code example shows the simulation of a track using the imported module and the built-in Brownian diffusion simulator.



from trait2d.simulators import BrownianDiffusion

params = dict()
params["Tmax"] = 0.5 # Maximum simulation time (s)
params["dt"] = 1e-4 # Simulation time resolution (s)
params["dL"] = 1e-12 # Simulation spatial resolution (m)
params["d"] = 1e-12 # Diffusion coefficient (m^2/s)
params["L"] = 1e-5 # Simulation domain size (m)
params["seed"] = 42 # Seed to initialize the random generator (for reproducibility)
params["quantize"] = False # Do not quantize the position to the spatial grid

simulator_brownian = BrownianDiffusion(**params)

simulator_brownian.run()


**Figure 3.**
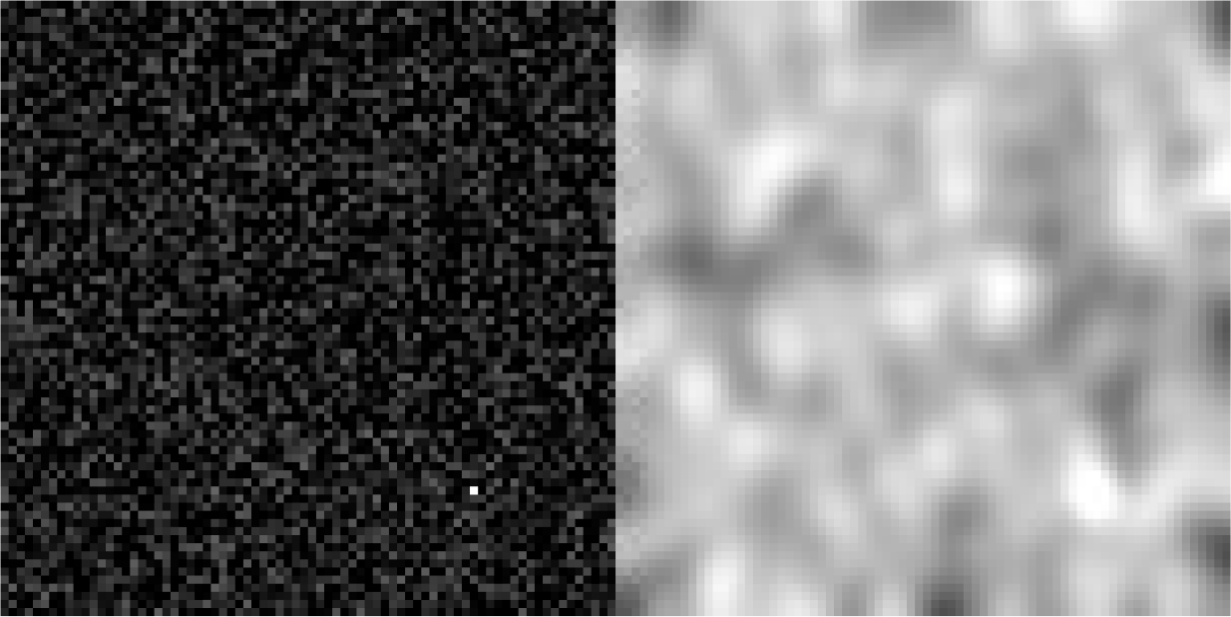
Simulated particle diffusion movie frame. (Left) Frame before and (Right) after the convolution by a PSF and the simulation of Poisson noise. An animated version of this simulation is available in this project Github repository.

The GUI also allows for the creation of an image sequence from the simulated track or a loaded trajectory file. Note that this is currently not possible from the imported module inside a Python script. Figure 3 shows such a simulated particle diffusion movie.

## Conclusion

TRAIT2D is a Python-based, open source and easily deployable toolkit for the analysis and simulation of two-dimensional SPT data. The combination of particle tracking, simulation, and trajectory analysis in the same package provides a number of benefits for the user. Another beneficial feature of this software is its user-friendliness for inexperienced users, thanks to the graphical user interfaces provided for each module. Nevertheless, the potential for customisation is retained and the scope of the module can be readily expanded by users with more advanced coding background.

## Data availability

The tracks used throughout the examples above are available in the source tree at the GitHub repository mentioned above in the examples folder: track1.csv, track2.csv, and track3.csv each contain a single track in the format expected by TRAIT2D. three_tracks.csv contains the same files which are identified by a separate id column. These example files report the trajectories obtained by performing particle tracking on three distinct timelapses of 40 nm diameter, streptavidin-coated gold nanoparticle linked to DSPE-PEG(2000)-biotin lipid analogues diffusing on an artificial membrane (Supported Lipid Bilayer) created on glass support. A track_example.tif file, consisting of a movie of a diffusing 40 nm streptavidin-coated gold nanoparticle linked to a biotinylated lipid analogue (DSPE-PEG(2000)-biotin), is provided as an example for the particle tracker module. The parameters_for_ track_example.csv provides an optimal set of parameter to track the particle in the aforementioned file, which is directly loadable in the tracker GUI, while the track_example_output.csv shows the outcome of the tracking operation.

## Software availability

• Software available from https://github.com/Eggeling-Lab-Microscope-Software/TRAIT2D/releases/ and https://pypi.org/project/trait2d.
• Source code available from https://github.com/Eggeling-Lab-Microscope-Software/TRAIT2D.
• Archived source code at time of publication: https://doi.org/10.5281/zenodo.4725268.
• License: GPL-3.0 License.
