# Supplementary material for: TRAIT2D: a Software for Quantitative Analysis of Single Particle Diffusion Data
Source: F1000Res. 2022 Jan 31;10:838. Originally published 2021 Aug 20. [Version 2] doi: 10.12688/f1000research.54788.2 (PMC8829092; doi:10.12688/f1000research.54788.2)
Supplement: Peer review history

# Peer review: Reviewer response for version 2

**DOI:** 10.5256/f1000research.119173.r121719

**Published:** 2022-02-09

**Authors:** Xavier Michalet

**Affiliations:** 1 Department of Chemistry and Biochemistry, University of California, Los Angeles (UCLA), Los Angeles, CA, USA; 2 California NanoSystems Institute, University of California at Los Angeles, Los Angeles, CA, USA

The authors have addressed the comments of my initial review, further increasing the value of their work. I have only a few additional comments, one about the science itself, the others about the new software version.

**1. Optimal number of points for MSD fits:**

 In their response, the authors write: “We acknowledge the fact that in the paper pointed out by the reviewer a rough way to estimate the optimal number of points is provided, which would prove useful in the analysis of Brownian diffusion”. That work does indeed provide a heuristic formula for the optimal number of points as a convenience, but the exact values are in the paper for everyone to peruse (they could in fact be stored as a precomputed array of values and change points), or they can be recalculated as described. The most important point is that prior knowledge of the relevant parameters (D and the localization uncertainty, sigma) is not needed to use it if the associated iterative numerical algorithm is used. A MATLAB script implementing this algorithm (as well as the unrelated but just as useful MLE approach introduced by Berglund) is provided as supporting information to this paper. There shouldn’t be any difficulty translating it in Python.

**2. Software tests:**

- 1 - trait2d_simulator_gui

After fixing a number of Python modules dependency errors (a step that requires minimal Python proficiency to overcome, but would surely discourage an unprepared user), I ran trait2d_simulator_gui and tried generating a trajectory using the default parameters (hopping diffusion). The file trait2d_simulator_gui error 1.txt reproduces the error message sent to the terminal. Similar errors were generated trying to simulate a “free diffusion” trajectory or a full image sequence.

 The error seems to reside in the self.seed=int(self.param_seed.get()) call and is apparently due to the default seed value, ‘None’. Using any integer value fixes the problem.

 As a note, generating an image sequence using the default parameters appears to require quite a bit of RAM and time on the fairly recent and powerful laptop I was using.

- 2 - trait2d_analysis_gui

After fixing a bunch of additional dependency errors, I was able to test the GUI using one of the trajectory files (.csv) in the examples folder. While the GUI seemed to be working fine, a bunch of additional errors were outputted in the terminal window (see trait2d_analysis_gui error 2.txt file).

- 3 - trait2d_tracker_gui

Once again, I had to fix some dependency issues before successfully launching the app. The example file was processed successfully using the default parameters.

 Summary:

- The installation problems I ran into are not insurmountable assuming some basic familiarity with the pip command but could result in a lot of overhead for the authors if they target non-Python savvy users. It might be useful to release some “standalone” versions (maybe PC and Mac only?) of the runtime to avoid these problems.
- I found the color contrast rather poor for the simulator and tracker GUIs. Consider using the same color scheme as for the analysis GUI for instance, although the graph part of it is also lacking contrast.

3. As a last point, I would suggest setting the correct option for ReadTheDocs such that a PDF version of the manual can be downloaded (web navigation is not always the best approach to discover a specific feature of a new software).

 ------------------------------------------------------------------------

 Content of trait2d_simulator_gui error 1.txt:

 c:\xxxxx\trait2d test\lib\site-packages\trait2d\simulators.py:279: UserWarning: mean displacement (2D) is more than half of average compartment length, results might be compromised

   warnings.warn("mean displacement (2D) is more than half of average compartment length, results might be compromised")

 Exception in Tkinter callback

 Traceback (most recent call last):

   File "c:\xxxxx\trait2d test\lib\tkinter\__init__.py", line 1892, in __call__

     return self.func(*args)

   File "c:\xxxxx\trait2d test\lib\site-packages\trait2d_simulator_gui\__init__.py", line 304, in generate_trajectory

     self.read_parameters()

   File "c:\xxxxx\trait2d test\lib\site-packages\trait2d_simulator_gui\__init__.py", line 391, in read_parameters

     self.seed=int(self.param_seed.get())

 ValueError: invalid literal for int() with base 10: 'None'

 ------------------------------------------------------------------------

 Content of trait2d_analysis_gui error 2.txt:

 c:\xxxxx\trait2d test\lib\site-packages\trait2d\analysis\models.py:22: RuntimeWarning: divide by zero encountered in true_divide

   return D + delta**2 / (2*t*(1-2*self.R*self.dt/t))

 c:\xxxxx\trait2d test\lib\site-packages\trait2d\analysis\models.py:22: RuntimeWarning: invalid value encountered in multiply

   return D + delta**2 / (2*t*(1-2*self.R*self.dt/t))

 c:\xxxxx\trait2d test\lib\site-packages\trait2d\analysis\models.py:34: RuntimeWarning: divide by zero encountered in true_divide

   return D_micro * (tau/t) * (1 - np.exp(-t/tau)) + \

 c:\xxxxx\trait2d test\lib\site-packages\trait2d\analysis\models.py:34: RuntimeWarning: invalid value encountered in multiply

   return D_micro * (tau/t) * (1 - np.exp(-t/tau)) + \

 c:\xxxxx\trait2d test\lib\site-packages\trait2d\analysis\models.py:35: RuntimeWarning: divide by zero encountered in true_divide

   delta ** 2 / (2 * t * (1 - 2 * self.R * self.dt / t))

 c:\xxxxx\trait2d test\lib\site-packages\trait2d\analysis\models.py:35: RuntimeWarning: invalid value encountered in multiply

   delta ** 2 / (2 * t * (1 - 2 * self.R * self.dt / t))

 c:\xxxxx\trait2d test\lib\site-packages\trait2d\analysis\models.py:48: RuntimeWarning: divide by zero encountered in true_divide

   D_micro * (tau/t) * (1 - np.exp(-t/tau)) + \

 c:\xxxxx\trait2d test\lib\site-packages\trait2d\analysis\models.py:48: RuntimeWarning: invalid value encountered in multiply

   D_micro * (tau/t) * (1 - np.exp(-t/tau)) + \

 c:\xxxxx\trait2d test\lib\site-packages\trait2d\analysis\models.py:49: RuntimeWarning: divide by zero encountered in true_divide

   delta ** 2 / (2 * t * (1 - 2 * self.R * self.dt / t))

 c:\xxxxx\trait2d test\lib\site-packages\trait2d\analysis\models.py:49: RuntimeWarning: invalid value encountered in multiply

   delta ** 2 / (2 * t * (1 - 2 * self.R * self.dt / t))

Are the conclusions about the tool and its performance adequately supported by the findings presented in the article?

Partly

Is the rationale for developing the new software tool clearly explained?

Yes

Is the description of the software tool technically sound?

Yes

Are sufficient details of the code, methods and analysis (if applicable) provided to allow replication of the software development and its use by others?

Yes

Is sufficient information provided to allow interpretation of the expected output datasets and any results generated using the tool?

Partly

Reviewer Expertise:

Single-molecule biophysics, single-photon detectors, fluorescence spectroscopy and imaging

I confirm that I have read this submission and believe that I have an appropriate level of expertise to confirm that it is of an acceptable scientific standard.

# Peer review: Reviewer response for version 1

**DOI:** 10.5256/f1000research.58301.r95432

**Published:** 2021-10-05

**Authors:** Eva Christensen Arnspang

**Affiliations:** 1 Department of Chemical Engineering, Biotechnology and Environmental Technology, University of Southern Denmark, Odense, Denmark

In this paper, the authors describe a novel analysis code for analyzing SPT data allowing for short-term observations. The code allows for tracking even at fast acquisition rates, yields information about different diffusion modes, and includes a GUI. A very well-written paper on novel SPT analysis method. I should be very interested in running the code in my own lab.

 The paper in its current form is acceptable for indexing, but it would enhance the reading experience for the reader with few well-chosen image examples of how the different components in the code improve the image analysis added, e.g. blurring, in addition to Figures 1 and 2. I would also recommend citing Mortensen *et al*., 2010 ^1^ - in this paper, a similar analysis technique is described.

Are the conclusions about the tool and its performance adequately supported by the findings presented in the article?

Yes

Is the rationale for developing the new software tool clearly explained?

Yes

Is the description of the software tool technically sound?

Yes

Are sufficient details of the code, methods and analysis (if applicable) provided to allow replication of the software development and its use by others?

Yes

Is sufficient information provided to allow interpretation of the expected output datasets and any results generated using the tool?

Yes

Reviewer Expertise:

Bioimaging, membrane proteins, nano domains, cell organelles, super-resolution microscopy

I confirm that I have read this submission and believe that I have an appropriate level of expertise to confirm that it is of an acceptable scientific standard.

## References

[1] : Optimized localization analysis for single-molecule tracking and super-resolution microscopy. *Nat Methods* .2010;7(5) : 10.1038/nmeth.1447 377-81 10.1038/nmeth.1447 20364147PMC3127582

# Response

**Published:** 2021-12-21

**Authors:** Francesco Reina

**Affiliations:** Leibniz-Institut für Photonische Technologien, Germany

We thank the reviewer for this kind report. We look forward to the applications of our code in her research group and remain open to suggestions for further changes, optimizations, and support requests.

 We are not sure what the reviewer intends when suggesting how the code would improve image analysis. In fact, the code provided does not act on the time-lapse image stacks themselves in any part, save from a simple background subtraction pre-processing step in the tracker GUI, and does not add any blurring on top. The blurring correction added refers to the fact that the localization of a diffusing particle, when imaged in its motion, will be subject to additional errors, which can be corrected with a term dependent on the sampling rate of the measurement, the diffusion coefficient, and a coefficient R dependent on the illumination mode used by the optical setup [1]. In case the reviewer intended something else, we could provide some examples upon suggestion.

 Concerning the additional reference, we appreciate the suggested work, which is definitely a worthwhile read for every scientist seeking a comparison of methods for accurate single-particle localization. Therefore, we have added the suggested reference with appropriate text modifications.

# Peer review: Reviewer response for version 1

**DOI:** 10.5256/f1000research.58301.r93050

**Published:** 2021-09-22

**Authors:** Xavier Michalet

**Affiliations:** 1 Department of Chemistry and Biochemistry, University of California, Los Angeles (UCLA), Los Angeles, CA, USA; 2 California NanoSystems Institute, University of California at Los Angeles, Los Angeles, CA, USA

**Summary:**

 This manuscript by Reina *et al*. describes a Python package implementing 1) single-particle tracking (SPT); 2) single-trajectory analysis (STA) by MSD (isotropic Brownian diffusion and isotropic anomalous diffusion) or ADC (Apparent Diffusion Coefficient, an acronym coined by the authors to designate a single-MSD point estimate of the diffusion coefficient); and 3) SPT movie simulation. The package is provided as a series of Python modules installed using the pip install command and can be run as 3 separate GUIs, as Python command lines, or within Jupyter Notebooks. The package comes with fairly detailed documentation (ReadTheDocs format, with code comments used to describe the different functions and classes in the module, and additional illustrated text description of the installation procedure and basic tutorials).

 The manuscript consists of a very basic introduction on the basics of SPT and single-trajectory analysis and a brief overview of a few examples of how to use the 3 different modules (SPT, Analysis and Simulation), and provides the necessary links to the Github repository and documentation. It is fairly well written and is sufficient to assess what the package will allow an inexperienced user to do. Basic attempts by this reviewer to test the different GUIs, followed by some failures or lengthy lists of warnings, seem to indicate that minor additional details and guidance could be useful for potential inexperienced users. The samples of code checked by the reviewer appear well-documented, but an overview of the relationship between different functions and classes might be useful for developers interested in using this project as a basis for their own tools (contributions do not appear to be discussed in the Github repository).

 The following are a few remarks which the authors could potentially use to remedy these deficiencies.

**Introduction**

 While this is not a review article, it seems that the introduction ignores a large body of literature and code on SPT and STA. For instance, the Particle Tracking Challenge paper (2014) ^1^ could be cited as it was an interesting effort to compare the multitude of isolated tools existing at the time, and would have provided a convenient set of test files to run the present software package on. There are many (literally dozens of) freely available packages to track and analyze single-particle data (easily found by an internet search with single-particle tracking as the keyword) and not all are using Matlab or “require licensed software to be executed” (Matlab can usually be replaced by the open-source Octave package), some are web-based (Spot-On) and many come with GUIs; therefore, some of the arguments put forward by the authors to justify their effort are somewhat artificial. This does not minimize the appreciable effort to document and simplify access to this type of tool for the novice user, although some additions might be needed (see later, GUI tests).

 Technically, the paper only scratches the surface of SPT and STA, but for obvious reasons since it is primarily a software presentation manuscript. However, since it appears, at least in part, targeted to novice SPT users, it would seem important to emphasize some important simplifications used in its algorithm implementations. For instance, the authors use the “Radial symmetry center approach” (which I was not aware of) because of its speed, but it is assuming radial symmetry, which might not always be appropriate (e.g. in the case of double-helix or astigmatic PSFs or simply long integration combined with fast diffusing particles). From a coding perspective, it could be useful to document how to replace this part of the code with another (or better yet, provide an example of such an optional alternative fitting algorithm – FluoBancroft comes to mind).

 When mentioning the blurring effect on diffusion analysis, the paper by Goulian & Simon is seminal, but in addition to Michalet 2010 already cited, Berglund 2010 ^2^, Michalet & Berglund 2012 ^3^ and Vestergaard 2012 ^4^ went some extra length to show how this affected the uncertainty on estimated parameters when performing optimal analysis (a question addressed further below). The authors might find it useful to point their readers to these papers (or not: it is not my intention to be self-serving, only to point out the often forgotten and rediscovered fact that this is not a completely trivial matter), which are a good starting point to follow the more recent literature on parameter estimation in more complex models.

**Trajectory analysis**

 MSD analysis, even with a large number of locations, is subtle (as just mentioned, see e.g. Fig. 7 of Michalet & Berglund 2012 ^3^), in the sense that the number of MSD points used has an effect on the precision of the extracted parameters. In fact, there is an “optimal” number (or range of numbers) to be used if one wants to minimize the error on the estimated parameters, and to some extent, this number can be automatically determined. Is that number the “Range” parameter found at the bottom of the Single-Track Analysis GUI window, and in that case, could an optional determination be offered? How is the user supposed otherwise to guess the best value without any theoretical insights?

 Anomalous diffusion analysis in the presence of an error is treated only in an ad-hoc manner in Eq. (8) (I could not find mention of it in ref. [30]) and it is therefore not quite clear how accurate the determination of either error, D_alpha or exponent alpha, is (not mentioning the eluded optimal number of points to use in the analysis). The point is, as discussed extensively in the recent literature, that MSD is almost certainly not an optimal method to analyze anomalous diffusion trajectories. I will not comment on the ADC approach, but clearly, the same comments would apply (optimal number of points and uncertainty on extracted parameters). In this respect, the “error” outputs on fitted parameters provided by the STA module are a bit mysterious (it would be a simple addition to write down their definitions in an appendix).

**Tracking**

 I would likewise recommend discussing (or cite a reference for) the LoG kernel and what its role is in image analysis. There are two user parameters to specify when implementing this kernel and it is unclear how to best choose them. In fact, the same question arises for the radial symmetry center algorithm (2 parameters) and the connection algorithm (2 parameters).

 All the previous discussions about tracking uncertainties, SNR, etc., seem for naught when contemplating the CSV file format for SPT trajectories, as it doesn’t include fields such as integrated intensity or localization uncertainty. Clearly, these parameters should be outputs of the SPT part and could/should be used to take into account as much possible information on the data as possible (for instance, to set the localization uncertainty to a fixed value?).

**Simulations**

 A priori, the correct simulation of diffusion with coefficient D is to use displacements distributed as N(0,sqrt(2D dt)) (see e.g. SI of ref. 10 ). The authors use instead sqrt(D dt) N(0, 1), which appears different based on the linearity of normal distributions (aN(0,s) + b = N(b,|a|s)). Please clarify.

 On P7, provide an explicit definition of the SNR, as there are several ways to define something that can play the role of SNR.

**Test of the provided code**

 I only tested the GUIs after installation in a dedicated Anaconda environment. Unfortunately, I encountered a few issues, which the authors might be able to address in the documentation.

trait2d_analysis_gui: Tested on the provided trackn.csv files, the terminal window spits out a large number of warnings.

- How is the user supposed to deal with those (and should they care)?
- What is the “Max. fit iterations” parameter for?
- What is the “Use parameters as initial guesses” checkbox for?
- What is the “Range” parameter for (it seems to be used to determine the number of data points (MSD or ADC) used for analysis)? I am assuming this is equivalent to the “fit_max_time” parameter discussed in the command line version (P 10) but again, this could be leaving a lot of freedom to a user unaware of the literature.

Trait2d_simulator_gui: Tested with default parameters and “Free Diffusion” resulted in errors in the Terminal window (for both Generate and Show Track). Same with Hopping Diffusion. It would be useful if the default parameters actually resulted in usable data. Minor tinkering resulted in viable results, but the generation of a simulated movie can take a considerable amount of time (and doesn’t seem to be parallelized); therefore a progress bar or some indication of the current frame number could be helpful. An indication of the resulting movie size could also be useful beforehand (those files can grow rapidly large when using the wrong parameters).

Trait2d_tracker_gui: It would be nice to provide an image stack example (and 8-bit TIFF stacks might be limiting in a world where most cameras output 16-bit images). I used the test image stack provided with TrackMate (a FIJI plugin) and the default parameters (with Light Spot option) but that failed (empty track). The file I generated by simulation using the previous module worked somewhat but it was difficult to figure out optimal parameters, and once tracking is started, it appears impossible to abort it (the progress was clearly indicated by the terminal output). Enabling partial initial processing of large stacks could prevent this kind of problem. Likewise, providing a short example stack – for instance, that discussed in the text – and initializing the GUI with appropriate parameters could help a user play around with parameters and understand the influence of each of the 7 important ones. Saving/loading analysis parameters for future reuse with similar datasets could also be useful.

**Typos:**

-  “otherDM”, “roundingDM”, “diffusion models DM”, “”command: DM” throughout the text.
- The sentence “allows the user to rely on objective parameters which diffusion model is more appropriate” does not make sense.
- P4: elucidate >> discuss.
- P6: The sentence “the condition that (x(t + dt)-x(t))^2 + (y(t + dt)-y(t))^2,…" does not make sense.
- P8: stored to the simulator >> stored in the simulator
- can be batched >> can be pooled
- stored to them >> stored in them
- at least a Python 3 >> at least Python 3
- P10: compatible to the analysis tool >> compatible with the analysis tool
- References: some of the journal volume numbers have 3 or 4 digits, which appears to be a mistake.

Are the conclusions about the tool and its performance adequately supported by the findings presented in the article?

Partly

Is the rationale for developing the new software tool clearly explained?

Yes

Is the description of the software tool technically sound?

Yes

Are sufficient details of the code, methods and analysis (if applicable) provided to allow replication of the software development and its use by others?

Yes

Is sufficient information provided to allow interpretation of the expected output datasets and any results generated using the tool?

Partly

Reviewer Expertise:

Single-molecule biophysics, single-photon detectors, fluorescence spectroscopy and imaging

I confirm that I have read this submission and believe that I have an appropriate level of expertise to confirm that it is of an acceptable scientific standard, however I have significant reservations, as outlined above.

## References

[1] : Objective comparison of particle tracking methods. *Nat Methods* .2014;11(3) : 10.1038/nmeth.2808 281-9 10.1038/nmeth.2808 24441936PMC4131736

[2] : Statistics of camera-based single-particle tracking. *Phys Rev E Stat Nonlin Soft Matter Phys* .2010;82(1 Pt 1) : 10.1103/PhysRevE.82.011917 011917 10.1103/PhysRevE.82.011917 20866658

[3] : Optimal diffusion coefficient estimation in single-particle tracking. *Phys Rev E Stat Nonlin Soft Matter Phys* .2012;85(6 Pt 1) : 10.1103/PhysRevE.85.061916 061916 10.1103/PhysRevE.85.061916 23005136PMC4917385

[4] : Optimal estimation of diffusion coefficients from single-particle trajectories. *Phys Rev E Stat Nonlin Soft Matter Phys* .2014;89(2) : 10.1103/PhysRevE.89.022726 022726 10.1103/PhysRevE.89.022726 25353527

# Response

**Published:** 2021-12-21

**Authors:** Francesco Reina

**Affiliations:** Leibniz-Institut für Photonische Technologien, Germany

We thank the reviewer for this extensive and constructive report. We acknowledged his comments, and have accordingly improved the manuscript, code, and documentation. Herein, we address all the reviewer’s concerns.

**Introduction**

 We appreciate the time and effort spent by the reviewer to analyse the introductory paragraph. Given the extensive body of literature on SPT data analysis, and with the good intention of not expanding the introductory paragraph too much, indeed some literature has been glossed over. We have included the Particle Tracking Challenge paper among the references, for a more comprehensive overview of work already carried out on SPT and STA data analysis.

 On the topic of why the tool has been developed, the reviewer's comment has sparked a constructive discussion among the authors, which led to a reframing of the tool in the context of Single Particle Tracking accessibility and the tools already available. This now reflects more accurately the intentions of the authors, and we thank the reviewer for their honesty that allowed us to further consider this point.

 We have highlighted limitations of the radial symmetry centre method and the reason it was selected among other existing methods. We also have proposed alternative solutions, providing general direction to replace the current sub-pixel localisation function in the code.

 We have chosen to address the choices of algorithm implementations as pointed out by this reviewer. In particular, as mentioned by the reviewer, we specifically addressed the choice to adopt the Radial Symmetry Center approach in order to fit the PSF of the diffusing particle in the movie as a deliberate decision originated by the research environment and the kind of data that favoured the inception of the present tool. Nevertheless, together with a general invitation to contribute to the code, a roadmap on how to implement a different tracking algorithm is provided.

 The short but comprehensive list of works suggested by the reviewer has been taken into account, and an invitation to further reading on the appropriate amount of points to take into consideration for the analysis has been added as a precaution to the correct use of the tool.

**Trajectory Analysis**

 Naturally, we concur with the reviewer that the number of MSD points, and indeed the model fitting methodology, have a significant effect on the precision of the parameters extracted, as this has been derived in several papers mentioned thus far in the reviewer report. The “Range” parameter, in the present tool, is a visual aid for the user to keep track of the time range considered in the model fitting. We acknowledge the fact that in the paper pointed out by the reviewer a rough way to estimate the optimal number of points is provided, which would prove useful in the analysis of Brownian diffusion especially, however, we deem this to be beyond the scope of this initial release. Nevertheless, given the open-source nature of the project, a contribution in this direction could also be implemented in the main code, and we rendered this process easier by adding specific guidelines for contribution (whose previous lack we thank the reviewer for pointing out). We, therefore, invite the reviewer to contribute an issue to our GitHub once the review process is complete so that the topic can be further debated and the tool developed in this direction.

 The inclusion of Ref [30] before equation (5) is an oversight by the authors and it has been amended, we apologize for the confusion. The inclusions of Eq. (5) and (8) are included as the MSD is still widely used as a measure to at least qualitatively evaluate anomalous diffusion [2], and thus its inclusion was deemed worthwhile. It should be noted, however, that while in the GUI, in the present release, both Eq. (7) and (8) are always fitted to the experimental data, this is not the case for a customized script including our library, which allows the user to freely exclude either, or define their own models altogether.

 Insofar as the accuracy of the determination of the parameters is concerned, we have added statements in the “Implementation” section detailing which algorithms in the SciPy library are employed and how the errors are thus calculated. Regarding the absolute accuracy of the method, we have emphasized the need for the user to further explore the topic, and we are sure that the present report, being publicly available, will also provide further guidance to novice SPT adopters.

 The comment on the ADC analysis regarding the errors of the estimated parameters was resolved by commenting on the model fitting procedure as for the MSD analysis. A reference to the range as intended by the reviewer is further added for the case of the free diffusion, in Eq. (11), since in this case it clearly would apply.

**Tracking**

 The reference to the Laplacian-of-Gaussian and a short discussion of its role is included in the manuscript, as well as the influence of the LoG kernel and threshold parameters into the spot detection. Suggestions on the parameter settings are also provided in the software manual.  We have added a link to the manual in the software interface to make it convenient for the user to find the information.

 We agree with the reviewer that localization uncertainty and integrated intensity can be a useful addition to the tracker output, and as a GitHub-based open-source project, we intend to extend the current version with a contribution from the community and our team. Regarding the current tool, the Radial Symmetry center approach in the implementation made available as a reference in the text only provides the standard deviation of the fitted 2D gaussian as an estimation, and not a calculated parameter, and does not include an integrated intensity. However, for the purposes of simple centroid determination, it provides an accurate and fast avenue. A cautionary statement regarding the use of the tool has been added to make the users aware of these characteristics of the tool.

**Simulations**

 The displacement distribution was modified to use N(0,sqrt(2D dt)). numpy.random.randn returns a sample from the standard normal distribution N(0,1). To convert this to any normal distribution N(mu, sigma^2) => sigma * N(0,1) + mu. For mu=0 and var=sigma^2=2D dt, we indeed need to change the multiplicative factor in the free (Brownian) equation from sqrt(D dt) to sqrt(2 D dt). This modification was done in both the software and in the paper.

 Also, in the context of the movie simulator, we used the SNR parameter as a way to adjust the contrast between the simulated particle intensity and the background intensity (contrast = particle intensity – background intensity). To better represent this, we have renamed this parameter to “contrast” in the GUI, the code, the documentation, and the paper. 

**Trait2d_analysis gui_gui:**

- The warnings produced by the analysis GUI were the result of an unchecked bug. This has been fixed and no warnings should be produced now. We would like to thank the reviewer for bringing this to our attention.
- The “Max. fit iterations” parameter is internally passed along to the “maxfev” (maximum function evaluations) parameter of scipy.optimize.curve_fit, which is responsible for performing the actual model fits. In case fits fail, it might help to increase this parameter.
- If the “Use parameters as initial guesses” checkbox is checked, the output parameters of the last fit will be passed to the “p0” parameter of scipy.optimize.curve_fit and used as initial guesses for the next fit. Similar to “Max. fit iterations” this might be used to improve existing fits in some scenarios.
- The Range parameter is, indeed, equivalent to the fix_max_time argument present in the code at various points. The issue of proper range determination is already presented in the paper. A link to the paper was added to the README file in the repository, so that it can serve as further documentation for the user, together with the present reviewer report. Once again, we extend an invitation to the reviewer to write an issue on the GitHub repository on this topic, following the finalization of the review process, to direct further developments of the tool.

**Trait2d_simulator_gui:**

 Thank you for these suggestions. We modified the default simulation parameters to make sure that the “Free diffusion” and “Hopping Diffusion” models can be used without necessitating too many resources. We added a dialog box to inform the user of the generated movie size before performing the movie simulation. Also, information about the simulation process is shown in the terminal.

**Trait2d_tracker_gui:**

 Taking into account the reviewer suggestions, we now have updated the tracker with the following features:

- Import 16-bit TIFF stack as well as 8-bit
- Save/load parameters of the tracker
- Test run of the linking algorithm, where the user can select the frame range to test the tracking before processing the entire image sequence. The detection results can be viewed directly in the viewer (preview button). 

We have also provided an example stack with a parameter file, which can be found in the Github repository. 

**Typos**

 We thank the reviewer for pointing these out for our attention. We addressed and corrected all of them as advised.

**References:**

 [1]     M. Goulian and S. M. Simon, “Tracking Single Proteins within Cells,” Biophys. J., vol. 79, no. 4, pp. 2188–2198, Oct. 2000, doi: 10.1016/S0006-3495(00)76467-8.

 [2]     G. Muñoz-Gil et al., “Objective comparison of methods to decode anomalous diffusion,” Nat. Commun., vol. 12, no. 1, 2021, doi: 10.1038/s41467-021-26320-w.
